# Indexed left ventricular mass to QRS voltage ratio is associated with heart failure hospitalizations in patients with cardiac amyloidosis

**DOI:** 10.1007/s10554-020-02059-1

**Published:** 2020-10-17

**Authors:** Jeremy A. Slivnick, Alexander L. Wallner, Ajay Vallakati, Vien T. Truong, Wojciech Mazur, Mohamed B. Elamin, Matthew S. Tong, Subha V. Raman, Karolina M. Zareba

**Affiliations:** 1 Division of Cardiovascular Medicine, The Ohio State University Wexner Medical Center, 473 W 12th Ave, Suite 200, Columbus, OH 43210 USA; 2 Division of Cardiology, The Christ Hospital Health Network, Cincinnati, OH USA; 3 Division of Cardiovascular Medicine, ProMedica Toledo Hospital, Toledo, OH USA; 4 Division of Cardiology, Indiana University School of Medicine, Indianapolis, IN USA

**Keywords:** Cardiac amyloidosis, Electrocardiography, Cardiac MRI, Cardiomyopathy, Heart failure

## Abstract

In cardiac amyloidosis (CA), amyloid infiltration results in increased left ventricular (LV) mass disproportionate to electrocardiographic (EKG) voltage. We assessed the relationship between LV mass–voltage ratio with subsequent heart failure hospitalization (HHF) and mortality in CA. Patients with confirmed CA and comprehensive cardiovascular magnetic resonance (CMR) and EKG exams were included. CMR-derived LV mass was indexed to body surface area. EKG voltage was assessed using Sokolow, Cornell, and Limb–voltage criteria. The optimal LV mass–voltage ratio for predicting outcomes was determined using receiver operating characteristic curve analysis. The relationship between LV mass–voltage ratio and HHF was assessed using Cox proportional hazards analysis adjusting for significant covariates. A total of 85 patients (mean 69 ± 11 years, 22% female) were included, 42 with transthyretin and 43 with light chain CA. At a median of 3.4-year follow-up, 49% of patients experienced HHF and 60% had died. In unadjusted analysis, Cornell LV mass–voltage ratio was significantly associated with HHF (HR, 1.05; 95% CI 1.02–1.09, p = 0.001) and mortality (HR, 1.05; 95% CI 1.02–1.07, p = 0.001). Using ROC curve analysis, the optimal cutoff value for Cornell LV mass–voltage ratio to predict HHF was 6.7 gm/m2/mV. After adjusting for age, NYHA class, BNP, ECV, and LVEF, a Cornell LV mass–voltage ratio > 6.7 gm/m2/mV was significantly associated with HHF (HR 2.25, 95% CI 1.09–4.61; p = 0.03) but not mortality. Indexed LV mass–voltage ratio is associated with subsequent HHF and may be a useful prognostic marker in cardiac amyloidosis.

## Introduction

Cardiac amyloidosis (CA) is a form of infiltrative cardiomyopathy in which misfolded amyloid proteins deposit within the myocardium, leading to progressive left ventricular hypertrophy (LVH), symptomatic heart failure (HF), cardiac arrhythmias, and death. The two most common proteins responsible for CA are transthyretin and immunoglobulin light chain. While previously thought to be a rare disorder, recent studies have demonstrated a high prevalence of CA in older patients with diastolic heart failure and low-flow, low-gradient aortic stenosis [1−4].

Unlike other disorders associated with increased wall thickness, LVH in CA occurs due to abnormal protein deposition rather than myocyte hypertrophy. Therefore, patients with CA often have disproportionately high LV mass relative to electrocardiographic (EKG) voltage. While low voltage is classically described in CA, this finding has poor sensitivity and may be absent in up to half of cases [5]. Additionally, the finding of low-voltage is not specific to CA either and may also be seen in other conditions including obesity, myocardial infarction, pulmonary disease, and pericardial effusion [6]. Studies have previously demonstrated that an increased LV mass to EKG voltage relationship (mass–voltage ratio) may be more specific to CA than low voltage alone [5, 7]. The LV mass–voltage ratio has been shown to reliably differentiate cardiac amyloidosis from other LVH-associated cardiomyopathies such as hypertensive heart disease and hypertrophic cardiomyopathy (HCM) [5, 7–9]. Additionally, increases in LV mass–voltage ratio appear to parallel disease progression in CA [10]. At this time, however, it is unclear whether LV mass–voltage ratio can be used to predict adverse outcomes in CA. Previous studies evaluating this have led to conflicting findings [11−13].

We sought to assess the relationship between LV mass–voltage ratio and the risk of HF hospitalization (HHF) and mortality in CA. We hypothesized that increased LV mass–voltage ratio would be predictive of adverse events in this patient population. We chose cardiovascular magnetic resonance (CMR) as it is the gold-standard non-invasive test to quantify LV mass [14].

## Methods

### Study population

We retrospectively studied 85 patients with cardiac amyloidosis who underwent comprehensive CMR exams and EKG between October 2010 and July 2019 at a single academic medical center. Patients were enrolled if they met the diagnostic criteria for cardiac amyloid which included a positive endomyocardial biopsy or a positive extracardiac biopsy with typical features of cardiac amyloidosis on cardiac imaging [15]. Typical cardiac imaging features were defined in accordance with expert consensus recommendations for multimodality imaging in CA [15]. Transthyretin CA was diagnosed with a positive endomyocardial biopsy, positive extracardiac biopsy with typical imaging features, or the presence of grade ≥ 2 Tc-PyP uptake in the absence of a monoclonal serum or urine light chain protein. Patients were considered to have immunoglobulin light chain CA if biopsy demonstrated AL fibrils either by immunohistochemistry or mass spectroscopy. Patients in whom EKG voltage could not be assessed due to excessive artifact or ventricular paced rhythm and those in whom LV mass could not be measured were excluded.

Clinical characteristics and comorbidities were established by review of the medical record. The following baseline clinical characteristics were collected: age, gender, ethnicity, height, weight, glomerular filtration rate (GFR), hematocrit, troponin, and brain natriuretic peptide (BNP). Information on comorbidities was queried from the medical chart including the presence of hypertension, diabetes, hyperlipidemia, and New York Heart Association (NYHA) Class. The Ohio State University Institutional Review Board approved this retrospective study and waived informed consent. This retrospective chart study involving human participants was in accordance with the ethical standards as laid down in the 1964 Declaration of Helsinki and its later amendments or comparable ethical standards.

### Electrocardiographic analysis

**Fig. 1:**
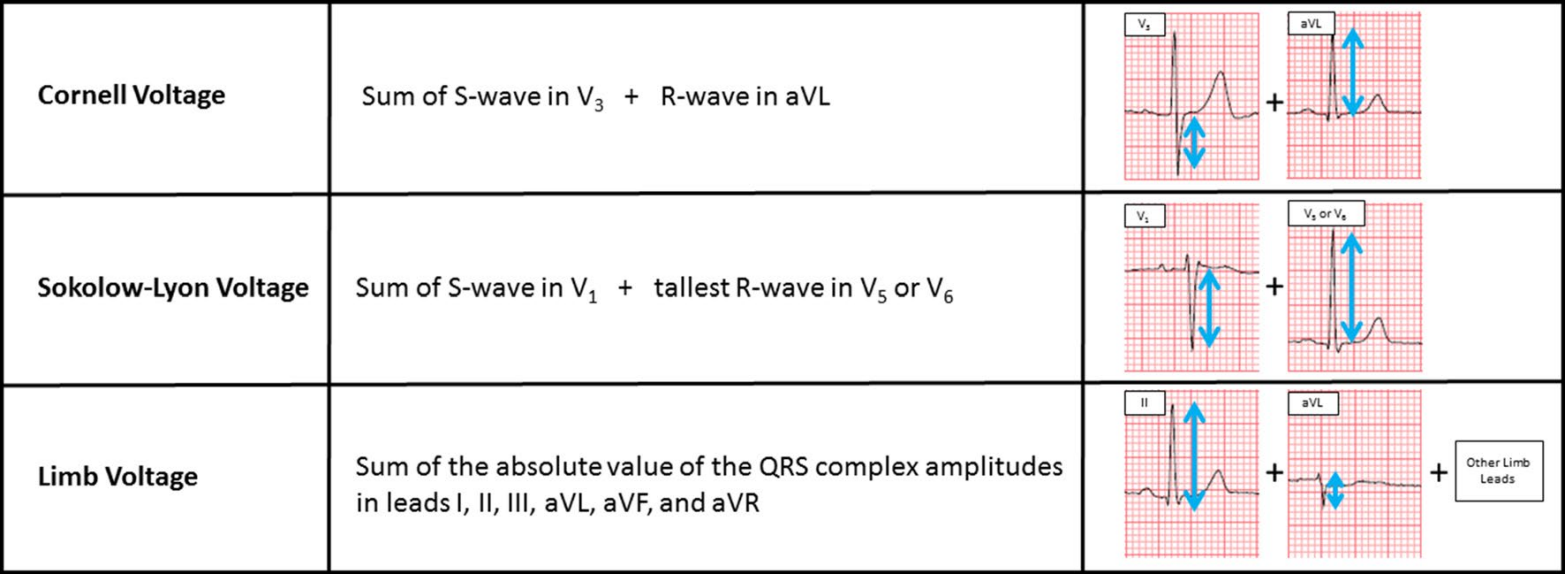
Electrocardiographic Voltage criteria: Definitions of voltage criteria utilized in the present study including Cornell voltage, Sokolow–Lyon voltage, and Limb voltage

Electrical parameters were assessed from a standard 12-lead EKG. Left ventricular voltage was calculated utilizing the Sokolow, Cornell, and limb voltage criteria which have been previously utilized in cardiac amyloidosis (Fig. 1) [9, 12]. The Cornell voltage was measured as the sum of the S-wave in lead V3 and the R-wave in lead aVL [16]. The Sokolow voltage was the sum of the S-wave in lead V1 and the tallest R-wave in leads V5 or V6 [17]. The limb voltage was measured as the sum of the peak-to-trough QRS voltage in all limb leads [12]. The indexed LV mass–voltage ratio was defined as the CMR-derived left ventricular mass indexed to body surface area (BSA) divided by the EKG voltage. Indexed LV mass–voltage ratio was calculated using all three voltage criteria described above.

### CMR protocol and analysis

All patients underwent clinical CMR scans with a 1.5 T scanner (Magnetom Avanto or Espree, Siemens Medical Solutions, Erlangen, Germany). Steady-state free precession sequences (SSFP) were used for assessment of ventricular volumes, ejection fraction (EF) and LV mass. Ventricular volumes and EF were measured from contiguous short-axis cine images using semi-automated software for endocardial segmentation using endocardial and epicardial contours at end-systole and end-diastole with Simpson’s rule. LV mass was calculated from the total end-diastolic myocardial volume multiplied by the specific gravity of the myocardium (1.05 g/ml) [18]. LV mass was then indexed to BSA.

Late gadolinium enhancement (LGE) imaging was performed using a gradient-echo inversion recovery sequence with magnitude and phase sensitive inversion recovery reconstructions 10 min after standard dose of gadolinium-based contrast agent [19]. The presence of LGE was assessed by 2 expert level 3 trained operators blinded to clinical data and had to be present in either two consecutive short axis slices or in two orthogonal imaging planes. MOdified Look-Locker Inversion Recovery (MOLLI) acquisition schemes were used to acquire T1 maps produced using vendor software before and 15 min after administration of contrast. T1 values and extracellular volume fraction (ECV) were measured and calculated utilizing interventricular septal values from the mid short axis view. The region of interest was placed in the mid myocardium with manual tracing to avoid partial volume effects [20, 21]. Myocardial ECV was calculated as previously described [22].

### Outcomes

The two co-primary outcomes, HHF and all-cause mortality, were assessed from the time of CMR as the first time point. HHF following CMR exam (regardless of any prior HHF) was identified by medical record review and defined as: physician documentation of HF along with symptoms (ie: dyspnea, orthopnea, paroxysmal nocturnal dyspnea) and physical signs (ie: edema, pulmonary rales) consistent with heart failure, and supporting clinical findings (ie: pulmonary edema, elevated BNP) with escalation in heart failure therapies [23]. Vital status was ascertained by Social Security Death Index queries and medical record review. Time to event was also recorded.

### Statistical analysis

Categorical data are presented as frequency with percentage, and comparison between groups was done using the chi-square test or Fisher exact test as appropriate. The distribution of continuous variables was assessed using skewness, kurtosis and visual inspection of the histogram. Continuous variables are presented as mean ± standard deviation (SD) for normal distribution or expressed as median (interquartile range) for non-normal distribution. Continuous variables were compared between 2 groups by t test or Mann–Whitney U test, as appropriate. Analysis of variance (ANOVA) or Kruskal–Wallis test was used to compare differences among more than 2 groups for normally and non-normally distributed variables, respectively. The optimal cutoff value for indexed LV mass–voltage ratio and outcomes of mortality and HF hospitalization was determined using receiver operating characteristic (ROC) curve analysis [24]. Multivariable cox proportional hazards model, adjusting for age, LVEF, New York Heart Association (NYHA) functional class, ECV and BNP, was used to assess the association between LV mass–voltage ratio and the aforementioned outcomes. Further, given that those patients who die cannot experience HF re-hospitalization, competing risk survival analysis using the Fine and Gray proportional subdistribution hazards model as a sensitivity analysis was used to examine the association between LV-mass–voltage ratio and hospitalization [25]. Additionally, as presence of the left bundle branch block (LBBB), and right bundle branch block (RBBB) can alter the amplitude of the QRS complex, a sensitivity analysis without RRBB and LBBB was performed to assess the association between LV mass–voltage ratio and adverse events. Proportionality assumptions of the Cox regression models were assessed by log–log survival curves and with the use of Schoenfeld residuals. The deviance residuals and the dfbeta values were used to examine influential observations. Hazard ratios are presented as mean and 95% confidence. A two-sided P- value of < 0.05 was considered statistically significant. The statistical analyses were performed using R software, version 3.6.1 (The R Foundation, Vienna, Austria).

## Results

### Demographics

A total of 85 patients were included in the study (mean age 68.8 ± 11.0 years, 22% female). Clinical characteristics are summarized in Table 1. There were 42 patients with transthyretin CA and 43 patients with immunoglobulin CA. Among those with transthyretin CA, 39 patients underwent genetic testing. Of these, 24(62%) patients had a germline mutation of the TTR gene while 15 (38%) had wild type transthyretin cardiomyopathy. In those with an identifiable genetic variant on genetic testing, the most common mutation identified was Val122, present in 18 (75%) patients. Of the patients with AL amyloid, 31 (65%) had received chemotherapy and 9 (19%) had undergone stem cell transplant. Of the patients with transthyretin CA, only 2 (5%) patients were on patisiran or tafamidis at the time of CMR. Compared with immunoglobulin light chain CA, patients with transthyretin CA were older and more likely to be African American. At median follow up of 3.4 years (IQR, 1.3–5.7 years), 42 (49%) patients were hospitalized for HF and 51 (60%) had died.

**Table 1:** Baseline clinical characteristics

	All (n = 85)	AL Amyloid (n = 43)	ATTR Amyloid (n = 42)	P value
Age, years	68.8 ± 11.0	64.0 ± 11.5	73.7 ± 8.1	** < 0.0001**
Female, n (%)	19 (22)	15 (35)	4 (10)	**0.004**
Race, n (%)				**0.0002**
White, n (%)	58 (68)	38 (88)	20 (48)	
Black, n (%)	25 (29)	4 (9)	21 (50)	
Other, n (%)	2 (3)	1 (3)	1 (2)	
Hypertension, n (%)	44 (52)	18 (42)	26 (62)	0.06
Hyperlipidemia, n (%)	45 (53)	22 (51)	23 (55)	0.74
Diabetes, n (%)	13 (15)	3 (7)	10 (24)	**0.03**
NYHA class, n (%)				0.35
I	6 (7)	5 (12)	1 (2)	
II	32 (38)	14 (33)	18 (43)	
III	38 (45)	19 (43)	19 (45)	
IV	9 (10)	5 (12)	4 (10)	
Body Mass Index, kg/m^2^	28.2 ± 7.1	27.4 ± 6.0	29.0 ± 8.1	0.31
Systolic blood pressure, mmHg	124 ± 22	117 ± 21	130 ± 21	**0.005**
Diastolic blood pressure, mmHg	72 ± 13	68 ± 12	76 ± 13	**0.003**
Creatinine, mg/dl	1.23 ± 0.49	1.21 ± 0.61	1.25 ± 0.34	0.70
GFR, ml/min/1.73 m^2^	70.3 ± 26.0	73.0 ± 30.5	67.4 ± 20.4	0.32
Hematocrit, %	37.6 ± 5.4	36.9 ± 5.7	38.2 ± 5.0	0.28
B-type Natriuretic Peptide, ng/L	459 (239–690)	583 (236–999)	428 (237–603)	0.08
Troponin, ng/dL	0.16 (0.07–0.24)	0.12 (0.05–0.23)	0.17 (0.07–0.25)	0.54

Bold variables are statistically significant

*GFR* glomerular filtration rate, *NYHA* New York Heart Association, *AL* immunoglobulin light chain, *ATTR* transthyretin

^*^Continuous variables are expressed as mean ± standard deviation or median (interquartile range). Categorical variables are presented as n (%)

### CMR characteristics

CMR characteristics for the whole cohort and individual amyloid subsets are demonstrated in Table 2. The mean indexed left ventricular mass was 104 ± 31 g/m^2^ and mean left ventricular ejection fraction was 46.5 ± 14.2%. Mean native myocardial T1 was 1105 ± 80 ms and mean ECV was 51.8 ± 10.9%. Compared to those with light chain CA, patients with transthyretin CA had higher indexed LV mass and max wall thickness but lower ejection fraction. Patients with transthyretin CA also had significantly higher ECV when compared to those with light chain CA.

**Table 2:** CMR characteristics

CMR data	All (n = 85)	AL Amyloid (n = 43)	ATTR Amyloid (n = 42)	P value
LV mass, g	200 (162–262)	176 (149–224)	232 (189–290)	**0.007**
LV mass index, g/m^2^	104 ± 31	97 ± 30	112 ± 32	**0.02**
Max Wall Thickness, mm	17.8 ± 4.3	16.7 ± 4.0	19.0 ± 4.4	**0.01**
LVEDVI, ml/m^2^	74.0 ± 18.3	68.5 ± 17.7	79.6 ± 17.3	**0.005**
LVESVI, ml/m^2^	40.3 ± 16.2	33.1 ± 12.0	47.6 ± 16.8	** < 0.0001**
Stroke volume index, ml/m^2^	33.4 ± 12.1	34.9 ± 13.6	31.9 ± 10.3	0.27
LVEF, %	46.5 ± 14.2	51.9 ± 13.1	41.0 ± 13.2	**0.0003**
LA volume index, ml/m^2^	62.1 ± 16.9	59.4 ± 15.7	64.9 ± 17.8	0.13
Native myocardial T1, ms	1105 ± 80	1090 ± 86	1121 ± 72	0.08
ECV, %	51.8 ± 10.9	48.5 ± 10.0	55.0 ± 10.9	**0.01**

Bold variables are statistically significant

^*^Continuous variables are expressed as mean ± standard deviation or median (interquartile range)

^†^LV, left ventricular; LVEDVI, left ventricular end-diastolic volume index; LVESVI, left ventricular end-systolic volume index; EF, ejection fraction; RVEDVI, right ventricular end-diastolic volume index; RVSVI, right ventricular end-systolic volume index; LA, left atrial; ECV, extracellular volume, *AL* immunoglobulin light chain, *ATTR* transthyretin

### Indexed LV mass–voltage ratio

The median time between EKG and CMR was 13 (2–35) days. Median voltage utilizing each of the criteria are presented in Table 3. The median indexed LV mass–voltage ratio by Cornell, limb, and Sokolow–Lyon criteria were 8.8 (4.9–12.9), 3.6 (2.3–5.5), and 9.4 (5.6–15.2) gm/mV/m^2^ respectively. There were no significant differences in LV mass–voltage ratio between the two amyloid subtypes by any of the three voltage criteria.

**Table 3:** EKG and Indexed LV mass–voltage data

	All (n = 85)	AL Amyloid (n = 43)	ATTR Amyloid (n = 42)	P value
Cornell voltage, mV	11 (8–19)	10 (7–15)	13 (9–21)	0.19
Mass–voltage ratio (Cornell criteria), gm/mV/m^2^	8.8 (4.9–12.9)	9.5 (4.9–13.3)	8.6 (4.9–11.8)	0.79
Limb voltage, mV	29 (20–43)	26 (18–38)	31 (23–46)	0.07
Mass–voltage ratio (Limb criteria), gm/mV/m^2^	3.6 (2.3–5.5)	3.6 (2.5–5.3)	3.2 (2.2–5.6)	0.80
Sokolow voltage, mV	11 (7–17)	9 (6–15)	13 (9–21)	**0.012**
Mass–voltage ratio (Sokolow criteria), gm/mV/m^2^	9.4 (5.6–15.2)	10 (6–18)	9 (5–13)	0.32
*Continuous variables are expressed as mean ± standard deviation or median (interquartile range)				

Bold variables are statistically significant

### Indexed LV mass–voltage ratio and outcomes

**Fig. 2:**
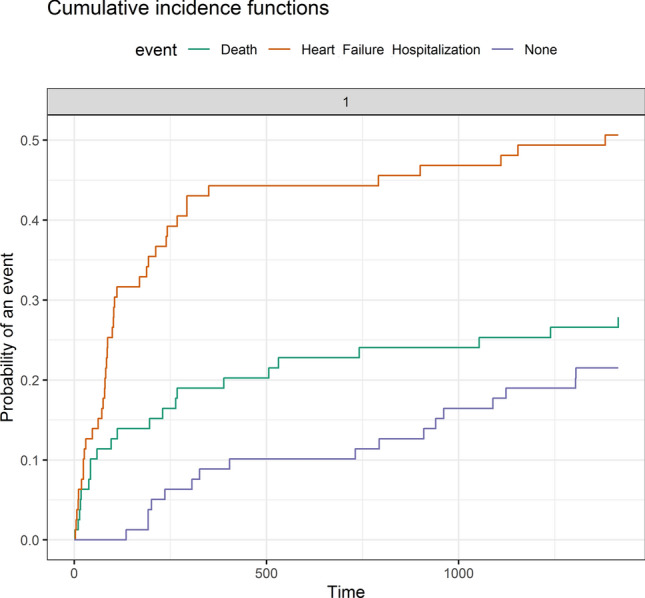
Cumulative Incidence of HHF and Death: Time to event analysis demonstrating the cumulative probability of event as a percentage of the whole cohort by the end of follow up. Death is denoted as green, heart failure hospitalization as brown, and no event as violet. *HHF* heart failure hospitalization

**Fig. 3:**
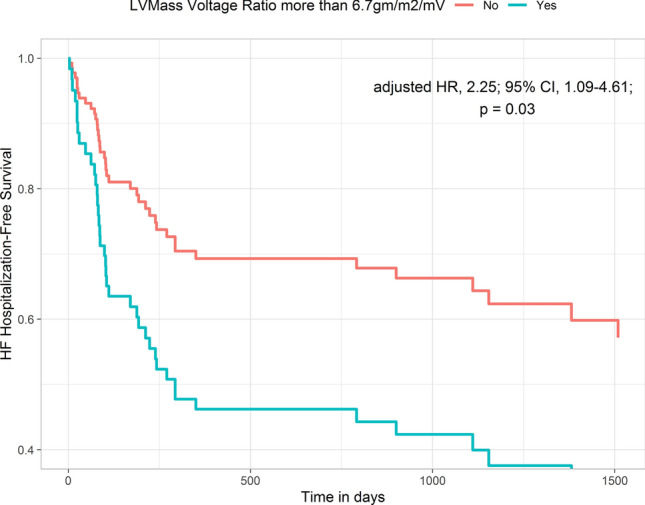
Heart Failure Hospitalization based on LV mass–voltage ratio: time to event analysis depicting rates of heart failure hospitalization amongst patients with and without indexed LV mass–voltage ratio > 6.7 gm/m^2^/mV after adjusting for age, BNP, and LVEF. LV mass–voltage ratio > 6.7 gm/m^2^/mV denoted as blue and ratio < 6.7 gm/m^2^/mV denoted as red. *LV *left ventricle; *BNP* B-type Natriuretic Peptide, *EF* Ejection Fraction

In unadjusted time to event analysis, LV mass–voltage ratio using Cornell voltage was significantly associated with both HF hospitalization (HR, 1.05 per 1 unit increase in mass–voltage; 95% CI 1.02–1.09, p = 0.001) and mortality (HR, 1.05 per 1 unit; 95% CI 1.02–1.07, p = 0.001). After adjusting for age, BNP, NYHA class, ECV and LVEF, the Cornell LV mass–voltage ratio was independently associated with HF hospitalization (HR, 1.06 per 1 unit; 95% CI 1.03–1.09,p < 0.001) but not all cause mortality (HR, 1.02 per 1 unit; 95% CI 0.99–1.05, p = 0.17) (Table 4). After excluding RBBB (7 patients) and LBBB (3 patients) from the analysis, LV mass–voltage ratio using Cornell voltage remained independently associated with HF hospitalization (HR, 1.06 per 1 unit; 95% CI 1.03–1.10,p < 0.001) but not all cause mortality (HR, 1.02 per 1 unit; 95% CI 0.98–1.05,p = 0.32). Fine and Gray proportional sub-distribution hazards model confirmed that Cornell LV mass–voltage ratio was associated with HF hospitalization with an estimated sub-distribution hazard ratio of 1.05 per 1 unit (95% CI 1.02–1.08, p < 0.001). The cumulative incidence of HF hospitalization and mortality as competing events are shown in Fig. 2. Using ROC curve analysis, the optimal cutoff value for Cornell LV mass–voltage ratio to predict HHF was 6.7 gm/m^2^/mV. A total of 52 (61%) patients had indexed LV mass–voltage ratio ≥ 6.7 gm/mV/m^2^. An LV mass–voltage ratio using Cornell ≥ 6.7 gm/m2/mV was strongly associated with HHF after adjusting for clinical covariates (HR, 2.25; 95% CI, 1.09–4.61; p = 0.03) (Fig. 3).

**Table 4:** Multivariable Cox-regression analysis for heart failure related hospitalization

	Model 1 (X^2^ = 18.77, p = 0.001)		Model 2 (X^2^ = 22.60, p < 0.001)		Model 3(X^2^ = 24.19, p < 0.001)	
	HR (95% CI)	p	HR (95% CI)	p	HR (95% CI)	p
Age	0.98 (0.96–1.006)	0.13	0.97 (0.94–0.99)	0.01	0.97 (0.95–1.00)	0.048
NYHA	1.66 (1.07–2.56)	0.02	1.63 (1.03–2.58)	0.04	1.47 (0.93–2.31)	0.10
BNP	1.00 (0.99–1.00)	0.44	1.00 (0.99–1.00)	0.25	1.00 (0.99–1.00)	0.31
ECV			1.05 (1.01–1.08)	0.008		
CMR LVEF					0.98 (0.95–1.00)	0.05
LV Cornell mass–voltage ratio	1.07 (1.03–1.10)	** < 0.001**	1.04 (1.001–1.08)	**0.04**	1.06 (1.03–1.10)	** < 0.001**

Bold variables are statistically significant

*LVEF* left ventricular ejection fraction, *BNP* B-type natriuretic peptide

In contrast, there was no significant independent association between LV mass–voltage by Sokolow (HR, 1.02 per 1 unit; 95% CI 0.99–1.06; p = 0.14) or limb voltage criteria (HR, 1.03 per 1 unit; 95% CI 0.90–1.18, p = 0.67) and HHF. Similarly, LV mass–voltage by Sokolow (HR, 1.03 per 1 unit; 95% CI 0.99–1.06; p = 0.16) or limb voltage criteria (HR, 0.99 per 1 unit; 95% CI 0.86–1.15; p = 0.95) was not associated with mortality in adjusted analysis.

## Discussion

**Fig. 4:**
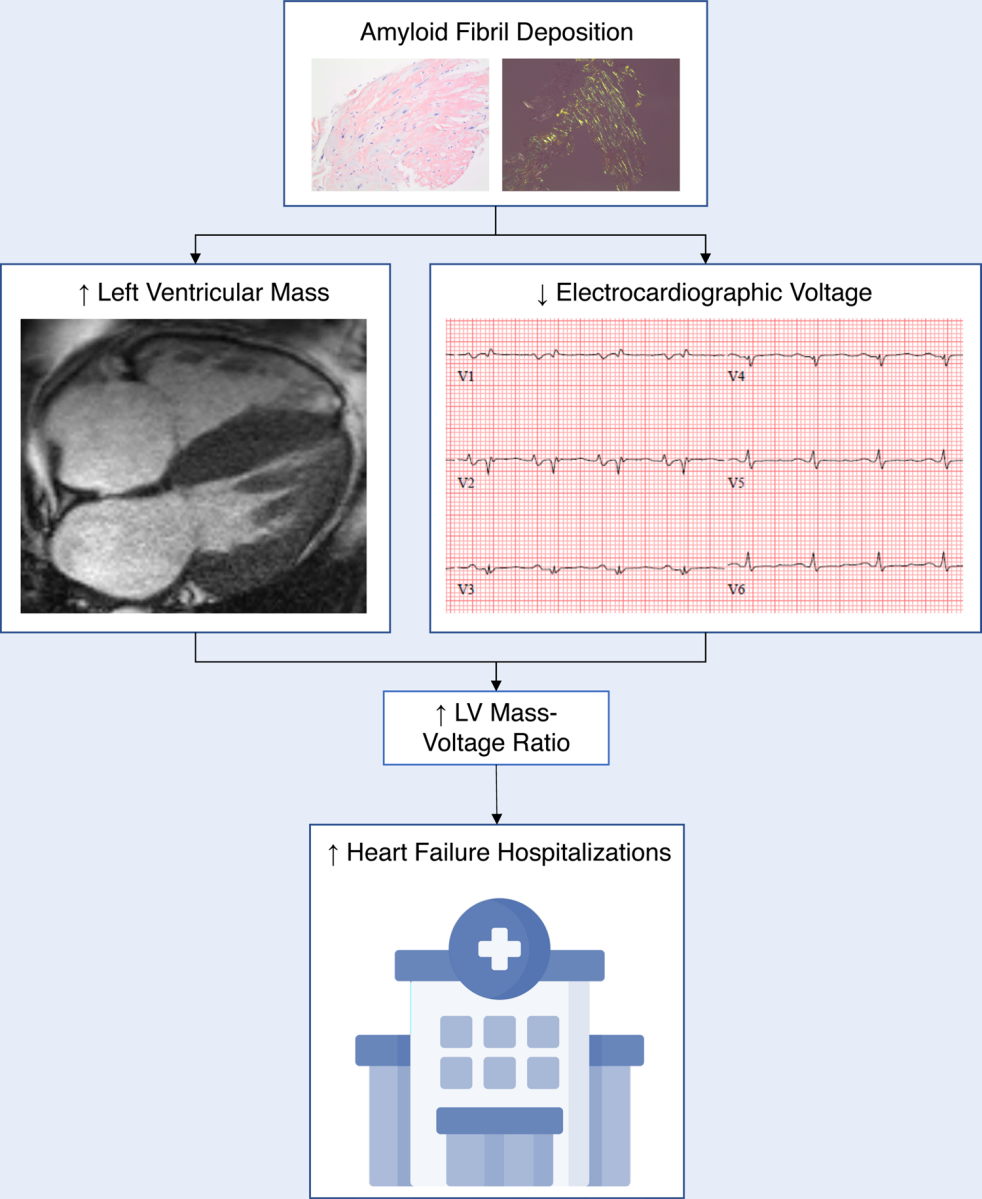
Indexed LV mass–voltage ratio reflects amyloid burden and is associated with HHF: Amyloid fibril deposition leads to increased left ventricular mass and decreased electrocardiographic voltage. Elevated LV mass voltage ratio is associated with heart failure hospitalization. *LV* left ventricle, *HHF* heart failure hospitalization

In this retrospective analysis, we report on the relationship between CMR-derived indexed LV mass–voltage ratio and clinical outcomes in patients with CA. We demonstrate a significant relationship between LV mass–voltage ratio and HHF even after adjusting for clinical covariates (Fig. 4). LV mass–voltage ratio was a better predictor of HHF than either LV mass or EKG voltage alone. LV mass–voltage ratio appears to account for both the hypertrophy and infiltration observed in CA while each single marker alone only accounts for one of these factors. Our findings parallel the Chang et al. study which demonstrated higher accuracy with echocardiography LV mass–voltage ratio for diagnosing CA when compared to EKG voltage alone [7].

Of the voltage methods, LV mass–voltage ratio by Cornell voltage criteria had the closest association with HF hospitalization. Several sensitivity analyses were performed to evaluate the robustness of the primary efficacy results. The association between elevated indexed LV mass–voltage ratio and risk of HF hospitalization was consistent across all sensitivity analyses.

The relationship between LV mass–voltage and clinical outcomes is not well described in CA. In a study by Cyrille et al., low voltage by Sokolow criteria was associated with a composite outcome of hospitalization, heart transplant or death [13]. However, they noted that neither LV mass as assessed by echocardiography or voltage-mass ratio were associated with outcomes in multivariate analysis. One possible explanation for this is that the quantification of LV mass with echocardiography is based on measurements made at a single slice of the left ventricular wall and relies on an assumption of normal left ventricular geometry. In contrast, CMR-derived LV mass is quantified using precise measurements of serial short axis slices which is less susceptible to measurement error [14, 26].

In our study, we also demonstrated an association between LV mass–voltage ratio and mortality on univariate analysis. However, this relationship did not reach significance when multivariate analysis was performed. It is possible that our study was underpowered for this outcome. Larger cohorts may be needed to best study the relationship between LV mass–voltage ratio and death in CA.

### Clinical implications

Based on our study, LV mass–voltage ratio as quantified by CMR appears to be a useful prognostic marker in patients with CA. Other CMR-derived markers including ECV and LGE have been consistently shown to predict adverse outcomes/survival in CA [27−29]. However, in our model, LV mass–voltage ratio remained a significant predictor of HHF even after controlling for ECV, suggesting that this marker may have independent prognostic value. Additionally, one limitation of ECV and LGE is that these markers require the administration of gadolinium contrast. Contrast use may not be possible in all patients such as those with advanced renal dysfunction or acute kidney injury. Native T1 relaxation time can be utilized in these patients but may not be available at all centers. LV mass–voltage ratio thus may play a complementary role to T1 mapping particularly in those patients who cannot receive contrast.

### Study limitations

This is a single center, retrospective study and results should be confirmed in a large multicenter cohort. Retrospective analysis was performed on patients referred for CMR thus introducing selection bias. Our study also lacked sufficient power to detect a difference in LV mass–voltage ratio and effects of treatment between transthyretin and immunoglobulin light chain CA. Lastly, ECV values were calculated from the mid short axis slice only as the basal T1 maps were not obtained at the time of the clinical scan, thus our data is representative of the mid-short axis slice.

## Conclusions

Indexed LV mass–voltage ratio as derived by CMR is associated with subsequent HF hospitalization. Indexed LV mass–voltage ratio may be a helpful prognostic marker in the care of patients with cardiac amyloidosis. Larger studies are needed to validate our findings, particularly in patients with less severe disease burden.

## Abbreviations

CA: Cardiac amyloid
SCT: Stem cell transplantation
LVH: Left ventricular hypertrophy
HF: Heart failure
HHF: Hospitalization for heart failure
EKG: Electrocardiogram
LV: Left ventricle
CMR: Cardiovascular magnetic resonance
Tc-PyP: Technetium pyrophosphate
BNP: B-type natriuretic peptide
GFR: Glomerular filtration rate
SSFP: Steady state free precession
RV: Right ventricle
ECV: Extracellular volume
SD: Standard deviation
ANOVA: Analysis of variance
ROC: Receiver operating characteristic
EF: Ejection fraction
NYHA: New York Heart Association
LGE: Late gadolinium enhancement
